# Comparative Analysis of Stable Gold Nanoparticles Synthesized Using Sonochemical and Reduction Methods for Antibacterial Activity

**DOI:** 10.3390/molecules28093931

**Published:** 2023-05-06

**Authors:** Mohammed Ali Dheyab, Azlan Abdul Aziz, Nazila Oladzadabbasabadi, Alyaa Alsaedi, Farhank Saber Braim, Mahmood S. Jameel, Asmeit Ramizy, Mohammad Alrosan, Ali Madi Almajwal

**Affiliations:** 1 Nano-Biotechnology Research and Innovation (NanoBRI), Institute for Research in Molecular Medicine (INFORMM), Universiti Sains Malaysia, Minden 11800, Penang, Malaysia; 2 Department of Physics, College of Science, University of Anbar, Ramadi 31001, Iraq; 3 Nano-Optoelectronics Research and Technology Lab (NORLab), School of Physics, Universiti Sains Malaysia, Minden 11800, Penang, Malaysia; 4 School of Science, STEM College, RMIT University, Melbourne, VIC 3000, Australia; 5 School of Industrial Technology, Universiti Sains Malaysia, Minden 11800, Penang, Malaysia; 6 Department of Biomedical Sciences, Cihan University-Erbil, Erbil 44001, Iraq; 7 School of Pharmaceutical Sciences, Universiti Sains Malaysia, Minden 11800, Penang, Malaysia; 8 Applied Science Research Center, Applied Science Private University, Amman 11937, Jordan; 9 Department of Community Health Sciences, College of Applied Medical Sciences, King Saud University, P.O. Box 10219, Riyadh 11433, Saudi Arabia

**Keywords:** gold nanoparticles, zeta potential, *S. aureus*, antibacterial activity

## Abstract

The increasing bacterial resistance and negative impacts of the present antibacterial agents have led to the search for novel antibacterial agents. This study focuses on the influence of synthetic methods on the aggregation stability and antibacterial activity of gold nanoparticles (NPs) prepared by using sodium citrate as a reducing and capping agent against *Staphylococcus aureus* (*S. aureus*). Gold NPs were synthesized using a simple and rapid sonochemical method and compared to gold NPs synthesized using a reduction method. The physicochemical features of gold NPs were characterized using UV-vis, XRD, TEM, and zeta potential, and the TEM results showed that the sonochemical method produced monodispersed spherical gold NPs with an average diameter of 18.5 nm, while the reduction method produced NPs with an average diameter of around 20 nm. The sonochemical method produced gold NPs with excellent stability (−48 mV) compared to the reduction method (−21 mV). The gold NPs with high stability also exhibited strong antibacterial activity against *S. aureus* present in water, indicating their potential use in water purification processes to limit bacterial growth. The outcomes of this research are expected to significantly contribute to the creation of new drugs by paving the way for the development of novel strategies to combat pathogens using highly stable gold nanoparticles. These gold NPs, produced via the sonochemical method, have the potential to be employed as beneficial nanocompounds in the medical industry.

## 1. Introduction

Nanotechnology is growing as a cutting-edge technology that involves numerous academic fields such as materials science, physics, chemistry, medicine, and biology [1]. Nanoparticles are typically less than 100 nm in size in each spatial dimension and are routinely synthesized by utilizing top-down and bottom-up approaches [2,3]. The top-down approach means reducing the size of the structure towards the nanoscale, while the bottom-up approach involves assembling large nanostructures from smaller atoms and molecules. The bottom-up technique is often used for the biological and chemical production of NPs, allowing for precise control over the size, shape, and composition of the resulting particles. NPs are particularly interesting for various applications due to their unique optical and physicochemical features, such as electrical conductivity, catalytic activity, chemical sensing, and medical diagnostic imaging [4,5].

Metallic NPs have demonstrated their versatility over the decades, owing to their adjustable size, structure, morphology, composition, and fascinating optical characteristics [6,7,8]. The literature contains well-documented production procedures for numerous types of metal nanoparticles [9,10,11,12,13,14,15]. Metallic nanostructures, particularly colloidal gold NPs, are used in biomedical applications due to their ease of production, simplicity, and speed of bioconjugation [16]. Gold NPs are known for their strong antibacterial activity, particularly against Gram-negative bacteria [17]. They are also recognized for their low cytotoxicity, high stability, and ease of synthesis using simple methods. When compared to other nanoparticles, AuNPs enable very simple surface modification, making it easier to generate new antibacterial combinations. Furthermore, AuNPs have great biocompatibility, which is a prerequisite for clinical applications [18,19]. While gold NPs have demonstrated antibacterial and anti-inflammatory properties, as well as the ability to enhance the effectiveness of certain drugs, more research is needed to determine their potential as a standalone therapeutic agent. Therefore, although gold NPs hold great promise, it is premature to conclude that they represent a solid alternative to existing therapeutics.

Noble nanomaterials are utilized for purifying water, which is one of the most important factors for supporting life on Earth. Water represents purity, health, uniqueness, and tranquility. Many diseases are caused by waterborne pathogens such as prions, viruses, fungi, protozoa and bacteria. The deactivation or removal of pathogenic bacteria in water is critical for maintaining water purity. Studies have demonstrated that NPs exhibit antibacterial properties against various pathogens [20,21,22,23]. To produce gold NPs with outstanding physicochemical properties, it is necessary to devise a novel methodology. Sonochemical synthesis is a commonly used method for creating nanomaterials because of the distinctive properties that can be obtained from acoustic cavitation [24]. Gold NPs have unique physical and chemical properties that make them attractive for biomedical applications, such as cancer diagnosis and therapy, drug delivery, and biosensors [25]. A sonochemical method is a promising approach for the synthesis of gold NPs with several advantages over conventional methods. First, the sonochemical method offers a high yield in the synthesis of gold NPs compared to other methods. Second, the sonochemical method can produce smaller NPs compared to other methods, which is important for biomedical applications. Third, the sonochemical method can produce monodispersed NPs with a narrow size distribution, ensuring the reproducibility of results and minimizing potential toxicity associated with size variations [26]. Fourth, the sonochemical method produces gold NPs with a high degree of biocompatibility, which is essential for their use in biomedical applications. Finally, the sonochemical method is a simple and easy-to-scale-up method for the synthesis of gold NPs. The method does not require toxic chemicals or organic solvents, and the NPs can be stabilized with biocompatible agents [27].

Acoustic cavitation produces a unique interface that combines energy and matter at extremely high temperatures, pressures, and cooling rates, resulting in a broad range of chemical reactions and the synthesis of a variety of high-quality nanostructures [10]. The implosive bubbles and shock waves created during acoustic cavitation produce high amplitude waves with a pressure exceeding 10 kbar. These bubbles collapsing can cause localized heating known as hot spots, which are different from those produced by traditional thermal processes [28]. Due to the unique interaction of energy and matter initiated by sonochemistry, this method is distinctive from other conventional methodologies and is more effective than direct coating.

However, the formation of spacious aggregates can result in the loss of antibacterial action [29,30], To prevent this, different polymers and surfactants are typically used to stabilize these metal colloids. Since stability is considered to be an essential characteristic of gold NPs dispersions, limited research has been conducted to investigate it as a parameter. Two types of protective strategies can be used to improve the stability of gold NP aqueous dispersions. Firstly, steric repulsion plays a stabilizing role by promoting the prompt adsorption of polymers and non-ionic surfactants at the interphase between phases [31]. The balance of repulsive and attractive forces is highly reliant on the adsorbent surface thickness, [32] which is reliant not only on the length of the chain but also on the adsorption mode of the polymer. The second mechanism of dispersing system stabilization investigated in this study is based on electrostatic repulsion. By introducing an ionic surfactant, the surface charge of the dispersed phase can be enhanced, providing electrostatic protection that enables the NPs to aggregate. The production technique can also affect the extent of aggregation.

Various investigations have utilized sodium dodecyl sulfate (SDS) as an anionic surfactant [33], as well as bromide or cetyltrimethylammonium chloride (CTAB and CTAC) as cationic surfactants [34]. Sodium citrate (Na_3_C_6_H_5_O_7_) was also used as a negatively charged or anionic surfactant in these investigations, and all three compounds have been identified as important stabilizing agents [6,35]. In NPs interactions with ionic surfactants, a possible mechanism of ionic molecule organization on the surface of NPs is described, in which the hydrophilic groups of the surfactant molecules are adsorbed on the NPs’ surface and the hydrophobic tails are directed outward to form the first layer. As a result, a counter-layer is oriented in the opposite direction, resulting in the interpenetration of the surfactant hydrophobic tails between the two layers, with hydrophilic groups pointing outward [36].

The current work aims to assess the impacts of the preparation method on the stability (zeta potential value) of gold NPs prepared by sonochemical and conventional reduction methods of the gold precursor (HAuCl_4_) with sodium citrate (Na_3_C_6_H_5_O_7_). The second goal is to assess the impact of gold NPs’ stability on antibacterial activity. As a result, the gold NPs obtained using the sonochemical approach demonstrated exceptional stability. The mechanism by which ionic surface interactions occur with gold nanoparticles and the reasons for their remarkable antibacterial activity will be explained.

## 2. Results and Discussion

### 2.1. Physicochemical Characterizations

**Figure 1:**
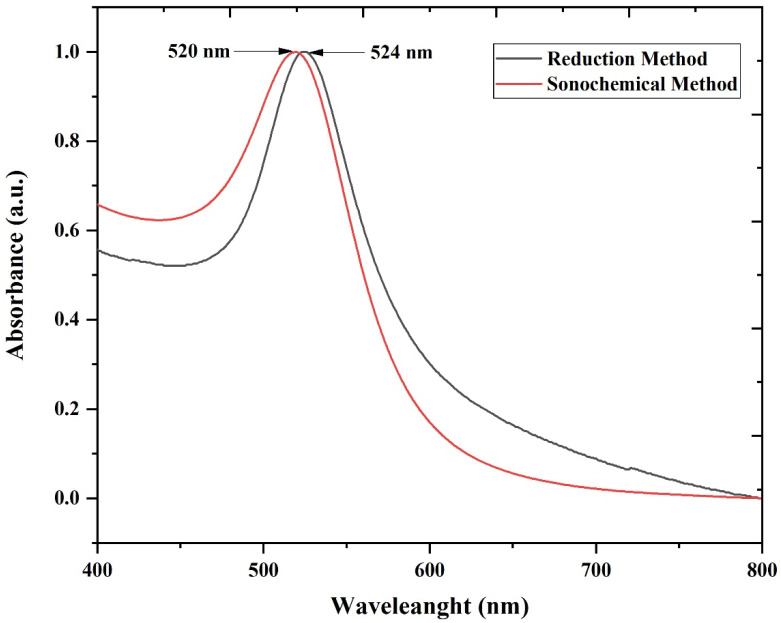
The absorption spectrum of gold NPs synthesized via the sonochemical method (red solid line) and reduction method (black solid line).

This section outlines the physicochemical examination of the produced gold NPs to evaluate their characteristics using various approaches. The production of gold NPs was verified by the characteristic bright red color, followed by UV-Vis examination. The UV-Vis technique is highly helpful for analyzing specific metal nanoparticles. Investigations of the surface plasmon resonance (SPR) band provided indirect evidence supporting the production of gold NPs. The SPR was measured between 400–700 nm under ambient conditions using deionized water as the reference standard [37]. The SPR peaks of gold NPs in the electromagnetic spectrum can be changed from visible to the infrared region [38]. The UV-Vis spectra (Figure 1) for gold NPs produced by the sonochemical technique and conventional method reveal a strong SPR at 520 nm and 524 nm, respectively, confirming the formation of gold NPs, which is consistent with the literature [39]. The application of the sonochemical method in the synthesis of AuNPs resulted in a plasmon peak that was significantly more noticeable compared to the conventional method. This observation highlights the sonochemical method’s ability to generate smaller and more uniform AuNPs compared to the conventional method, as evidenced by the significantly more noticeable plasmon peak. In contrast, the SPR peak observed under the conventional method was found to be at approximately 524 nm, with slight broadening due to the larger size of the synthesized AuNPs. The broadening and redshift of the SPR peak can be attributed to an increase in the number of electrons, which is directly proportional to the volume of the AuNPs. This increase in the extinction of AuNPs causes the SPR peak to broaden. When the size of the AuNPs approaches the wavelength of light, the metal nanoparticles become less homogeneous and polarized by incident light. As a result, there is an increase in radiative damping, leading to an increase in the scattering contribution and plasmon line width [40]. The use of the sonochemical method in synthesizing AuNPs is a promising technique due to its ability to generate smaller and more uniform nanoparticles, as seen in the narrower and more significant plasmon peak. These findings contribute to the development of more efficient and reliable methods for synthesizing nanoparticles with desired properties.

**Figure 2:**
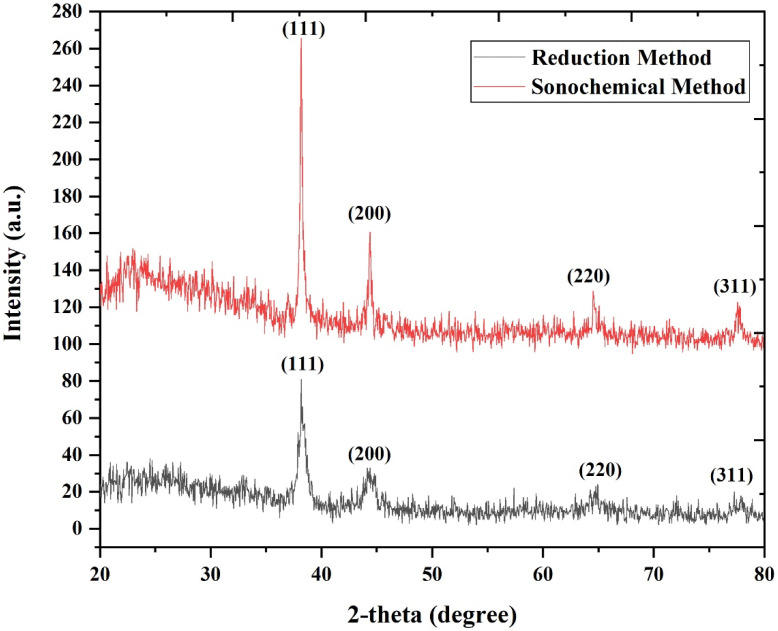
The XRD pattern analysis reveals the preparation of gold NPs with a face-centered cubic structure synthesized using the sonochemical process (red solid line) and the reduction method (black solid line).

The formation of gold NPs using the sonochemical and conventional methods was tested by XRD analysis, as shown in Figure 2. The main four peaks at 2θ values for both samples are illustrated in Table 1. The graph clearly shows that both samples’ XRD patterns have four distinct peaks that correspond to the (100, 200, 220, and 311) planes of gold NPs, confirming the high crystallinity of gold NPs with a cubic inverse spinel structure. The positions and relative intensities of the diffraction peaks match well with those of the gold, which is confirmed by the JCPDS card no. 01-089-3697 space group = Fm-3 m [41,42]. The absence of any extra peaks of other phases of NPs in the XRD spectra indicates that the produced NPs are of high purity. According to a published study, the presence of particles during cavitation contributes to a shift in the symmetry of bubble degradation, which can result in the formation of various NP frameworks under different ultrasound wave conditions [43]. In addition, ultrasound waves have significant mechanical effects, besides chemical waves. The implosive nature of cavitation bubbles generates shock waves that can damage the surfaces of affected NPs, thereby impacting their morphology and crystal structure. As bubbles implode, cavitation can also result in the formation of an extremely turbulent flow throughout the liquid mixture in the absence of particles. Therefore, ultrasound waves produced during cavitation can result in the formation of diverse NP frameworks and affect the crystal structure and morphology of gold NPs [44].

**Figure 3:**
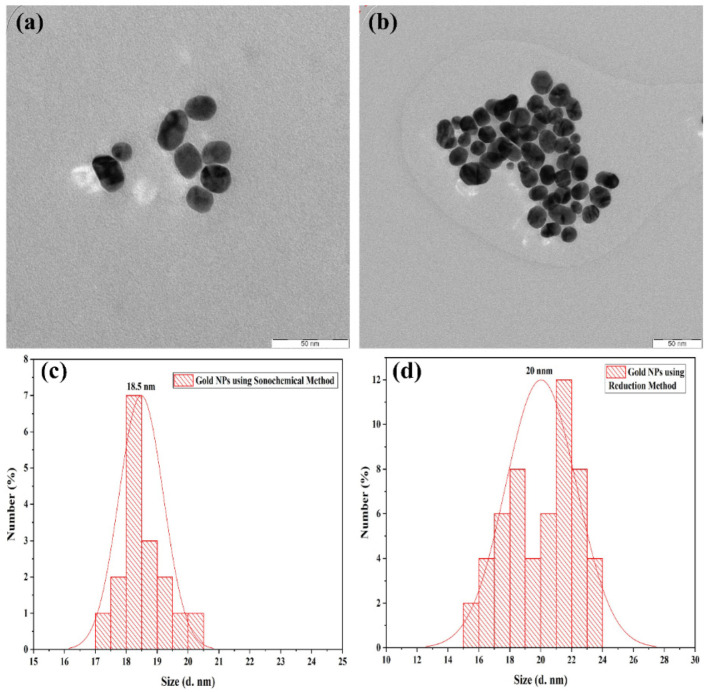
The TEM images and size distribution of semi-spherical gold NPs are shown with a scale bar of 50 nm in (**a**,**c**) obtained via the sonochemical method, while (**b**,**d**) display those obtained via the reduction method.

For better identification of the effect of the sonochemically assisted preparation and reduction method by using citrate as a reducing agent, in terms of the shapes and size of the colloidal gold NPs, the samples were evaluated via transmission electron microscopy (TEM). The TEM images of the gold NPs are depicted in Figure 3a,b. The TEM images revealed that the sonochemically assisted synthesis and reduction method resulted in well-uniformed spherical gold NPs with an average size of 18.5 nm and 20 nm, respectively. The size and shape of the NPs can have a significant impact on their properties and potential applications, making it important to accurately characterize them. The uniform spherical shape and relatively small size observed in the TEM images of the synthesized gold NPs indicate that they have great potential for biomedical and environmental applications.

**Figure 4:**
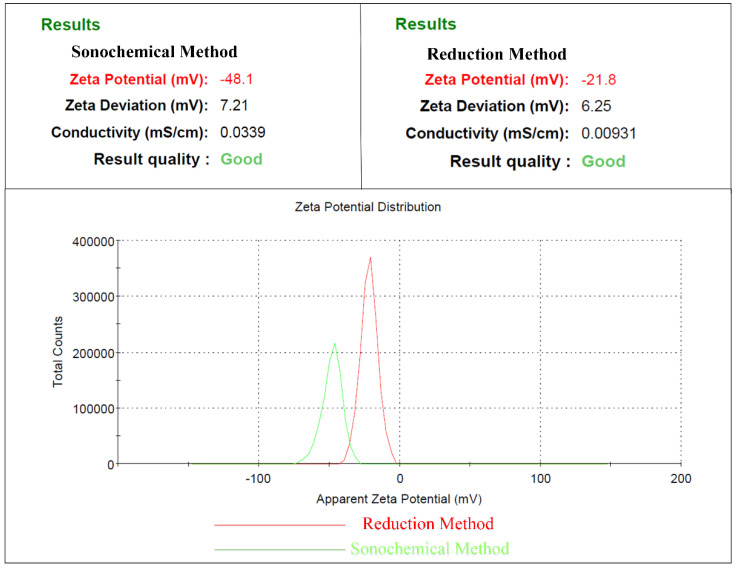
The zeta potential distribution (mV) of gold NPs synthesized through the sonochemical method is shown as a green solid line, while that of NPs synthesized via the reduction method is shown as a red solid line.

Gold NPs have a high specific surface area that makes them resistant to agglomeration. Zeta potential analysis provides information on the surface charge of particles and their physical stability in suspensions. A high positive or negative zeta potential value indicates that the NPs repel each other and are less likely to agglomerate. However, there is no way to prevent the clumping of NPs with poor zeta-potential values, indicating a significant flocculation propensity. A general criterion for determining stable and unstable solutions is often ±30 mV. Particle suspensions with zeta potentials greater than 30 mV are considered stable [35]. The zeta potential value of gold NPs synthesized using the sonochemical method is highly electronegative (−48.1 mV) due to the complete capping action of citrate ions, and particle-particle repulsion prevents particle aggregation or precipitation (Figure 4) [45]. In contrast, the zeta potential value of gold NPs synthesized using the conventional method is −21.8 mV, which is much lower than that of gold NPs synthesized using the sonochemical method. The sonochemical method functionalizes the structure and surface of the NPs, preventing particle accretion and stabilizing aqueous solutions of gold NPs [46,47]. Therefore, it is important to define the precise sonochemical conditions for various nanofluids since different nanofluids can exhibit a variety of dispersion behaviors using sonic energy. Previous studies have demonstrated that directly dispersing NPs in the base fluid using sonication (horn/probe) is more effective than conventional methods due to different Interactions between energy and matter [48]. The sonochemical technique allows for a variety of chemical reactions and the production of unique nanostructures [49]. However, the local environment can influence the acoustic cavitation dynamics, and the acoustic cavitation phenomenon can produce implosive bubbles and shock waves that result in pressures exceeding 10 kbar [50]. The ultrasound-induced phenomenon enhances the attachment of ions or molecules to particles, which explains the enhanced stability of gold NPs generated using the sonochemical approach [51].

### 2.2. Antibacterial Activity of Gold NPs

**Figure 5:**
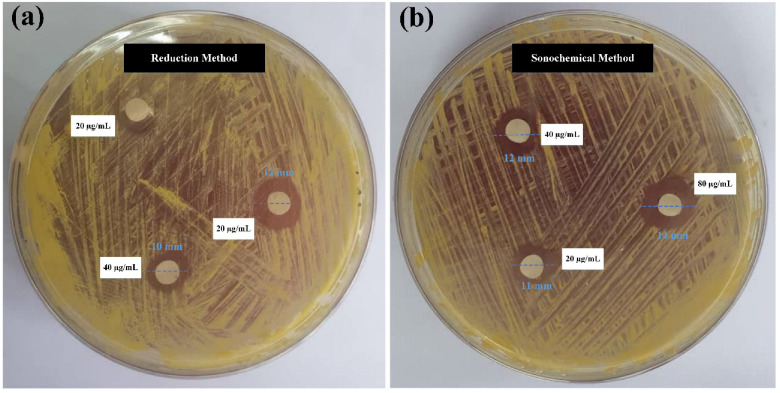
Antibacterial activity against *S. aureus* of gold NPs synthesized through (**a**) the reduction method and (**b**) the sonochemical method.

The antibacterial properties of the synthesized gold NPs solution against *S. aureus* were studied using the disc diffusion technique. Three sterile Whatman paper discs with a diameter of 5 mm and containing 20, 40, and 80 µg/mL of gold NPs were placed individually on the NA agar plate. The diameter of the inhibitory zone (in mm) was evaluated after 24 h of incubation at 37 °C. The inhibition activity was measured in relation to the diameter of the inhibition zone. The results are given in Figure 5a,b. It was discovered that at 20, 40, and 80 µg/mL concentrations of gold NPs synthesized by the sonochemical method, the inhibition zone diameter was 11, 12, and 14 mm against *S. aureus*, respectively, while for the same concentrations of gold NPs synthesized by the reduction method, the inhibition zone diameter was 0, 10, and 12 mm, respectively. The finding in Figure 5 demonstrates that the high-stability gold NPs exhibited the highest antibacterial activity. In addition, the lowest concentration of gold NPs synthesized by the reduction method did not inhibit the bacterial growth zone, while the same concentration of gold NPs synthesized by the sonochemical method inhibited the growth zone of bacteria due to the high stability of this sample (−48.1 mV).

The maximum antibacterial activity is directly related to the best surface stabilization (highest stability). In addition to the unique surface areas and facet reactivity of gold NPs, sonochemical methods can help to improve antibacterial activities by enhancing the stability of gold NPs and therefore increasing the permeability of the cell wall or by breaking the cell wall of bacteria [52]. As a result of their mobility and significant surface energy, non-aggregated NPs can strongly interact with the cell wall, which is not diminished by the development of large aggregates. These NPs have shown great potential for infections due to their unique physicochemical properties. Gold NPs, in particular, have been extensively studied and found to be highly biocompatible, stable, and easily functionalized with various targeting molecules. Therefore, gold NPs are a promising candidate for the development of antibacterial agents.

### 2.3. Mechanisms of Action

**Figure 6:**
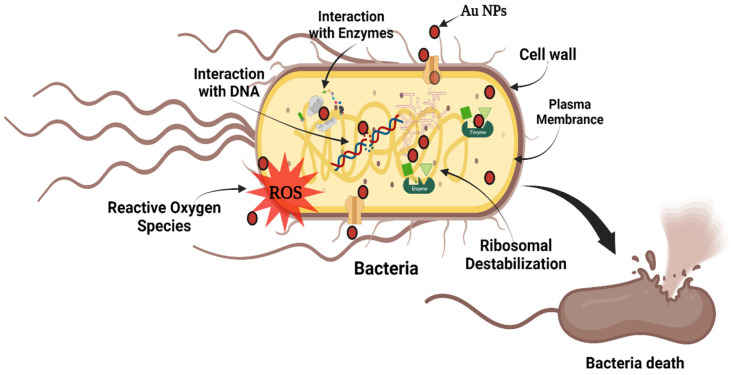
The possible mechanisms of antibacterial activity for gold NPs.

Biophysical interactions between nanoparticles (NPs) and bacteria have been shown to occur via biosorption, aggregation, and cellular absorption, leading to toxicity and membrane degradation. Figure 6 shows the possible mechanisms of antibacterial activity for gold NPs. A complete understanding of antibacterial processes is still necessary to enhance the potency of NPs in treating diseases [53]. Because of the extraordinary antibacterial and anticancer characteristics of gold NPs, the production of these nanoparticles has piqued the interest of many researchers over the years. The antibacterial activity of gold NPs is mostly due to their high surface-area-to-volume ratio, which allows for more atoms to exist on the surface and, as a result, greater interaction with the environment. Moreover, the nanosized and stability of these particles allow them to pass through cell membranes, interact with intracellular components, and eventually cause cell death during the multiplication phase.

The mechanism involved in the antibacterial activity has been suggested to be dependent on the gold NPs’ size, surface modification, and bacterial strain [54]. Some observations may be made to explain how gold NPs destroy bacteria. Gold NPs cause vesicle production, which results in membrane perforations [55]. Gold NPs increase the concentration of intracellular reactive oxygen species (ROS) in *S. aureus* [56]. Gold NPs could potentially bind to bacterial DNA, leading to inhibition of DNA uncoiling and transcription, ultimately promoting bacterial death [57]. It is noteworthy that gold NPs have the ability to traverse the bacterial surface and form a spherical cytoplasmic structure called an inclusion body of gold NPs [58]. This is an atypical subcellular structure formed due to the interaction with the gold NPs, the composition of which remains unidentified. A strategy was devised to enrich the gold NPs and evaluate their constituents. The proposed mechanism for gold NPs’ antibacterial activity is associated with the formation of ROS, which results in an increase in oxidative stress in microbial cells and the release of intracellular lactate dehydrogenase enzyme into the extracellular medium in the form of vacuole formation as an indicator of potent activity [59,60].

## 3. Materials and Methods

### 3.1. Materials

The chemical reagents sodium citrate (Na_3_C_6_H_5_O_7_) and chloroauric acid (HAuCl_4_·4H_2_O) were used as precursors to synthesize the gold NPs. They were bought from Sigma-Aldrich and used directly without further purification. All the chemicals required for the synthesis were obtained from Sigma Aldrich, Saint Louis, MO, USA. *S. aureus* bacteria were purchased from Biomedia SPD Scientific, Singapore.

### 3.2. Characterization of Au NPs

XRD patterns of the gold NPs were acquired at room temperature by means of an X-ray diffractometer (PANalyticalX’pert PRO MRD PW 3040, Malvern, Enigma Business Park, UK) with CuKa (λ = 1.54050 Å) to investigate their crystalline structure. The size of the sample was derived using transmission electron microscopy (TEM, Zeiss Libra 120, Oberkochen, Germany) at 100 kV. ImageJ software (1.52 v) was used to measure the particle size distribution of the sample. The absorption properties of the gold NPs were determined using a UV-Vis-NIR spectrophotometer (UV-3600, Shimadzu, Columbia, MD, USA). To assess the stability of the gold nanoparticles, dynamic light scattering (DLS) was performed using a ZETASIZER Nanoseries Model ZEN 3600 (Montgomeryville and York, PA, USA) instrument from Malvern Instruments to measure their zeta potential.

### 3.3. Ultrasonic Synthesis of Gold NPs

The sonochemical technique was used to produce the gold NPs. A vibra-cell ultrasonic solid horn with a tip size, frequency and power output of ½ inch, 20 kHz and 750 watts respectively was used in the gold NPs’ synthesis [61]. A gold precursor (HAuCl_4_) was dissolved in deionized water. In one pot, the solution of gold precursor (0.03 M, 1 mL) and an aqueous solution of sodium citrate (Na_3_C_6_H_5_O_7_) was mixed in distilled water (0.03 M, 10 mL). Following that, a high-density ultrasonic probe operating at 20 kHz via an ultrasonic horn was immersed in the solution for 5 min to generate the gold NPs. A few of the aqueous colloidal NPs were dried at 90 °C for 24 h to evaluate the crystalline structure of the gold NPs.

### 3.4. Conventional (Reduction) Synthesis of Gold NPs

A gold precursor (HAuCl_4_) was dissolved in deionized water. In one pot, the solution of gold precursor (0.03 M, 1 mL) and an aqueous solution of sodium citrate (Na_3_C_6_H_5_O_7_) was dissolved in distilled water (0.03 M, 10 mL). This mixture was continuously agitated at 700 rpm indefinitely before being stored at 100 °C. After 20 min, the color of the liquid changed from yellow to red wine, indicating that the nucleation process had begun.

### 3.5. Antibacterial Performance Assays

The antibacterial activity of the synthesized gold NPs against Gram-positive *S. aureus* was tested in vitro. Overnight cultures were streaked onto NA agar plates. Sterile Whatman paper discs of 5 mm diameter, each holding 10 μL of gold NPs synthesized by the sonication and reduction methods (80, 40, and 20 μg/mL), were placed in a serial order on each plate. The diameter of the inhibitory zone (in mm) was measured after 24 h of treatment at 37 °C. The clear diameter of the inhibition zone was used to assess the bactericidal action. The wider the inhibitory zone, the greater the antibacterial action.

## 4. Conclusions

In this study, the successful synthesis of gold NPs using citrate as a reducing agent through sonochemical and reduction procedures was demonstrated. The gold NPs were characterized using various analytical techniques including UV-vis, TEM, XRD, and DLS. The sonochemical procedure was found to be more efficient than the reduction method, requiring less time and being more straightforward. The TEM analysis of the sonochemical sample showed that the gold NPs were spherical in shape, with a narrow size distribution and an average size of 18.5 nm. The XRD analysis confirmed that the synthesized gold NPs were highly crystalline. Moreover, the DLS analysis revealed that the sonochemical method provided greater stability to the gold NPs than the reduction method, with a zeta potential of −48 mV compared to −21 mV. The antibacterial activity of the synthesized AuNPs was evaluated against *S. aureus*. The results showed that the sonochemically synthesized AuNPs exhibited greater and moderate antibacterial activity against *S. aureus* than the reduction method. The improved binding and stability of the gold NPs were thought to be the reasons behind their tunable antibacterial activity against *S. aureus*. Overall, the study provides important insights into the potential use of gold NPs as antibacterial agents against *S. aureus*, which is a major public health concern. The sonochemical method of synthesis was found to be more efficient and straightforward, making it a promising approach for the large-scale production of gold NPs for biomedical applications.

## Figures and Tables

**Table 1:** X-ray data for gold NPs synthesis using both methods.

Sample	hkl	2Ө^o^	D (nm)	Intensity (a.u)
(JCPDS card no. 01-089-3697 space group = Fm-3m)	111	38.17	2.35	100%
	200	44.37	2.03	45.4
	220	64.56	1.44	23.9
	311	77.54	1.23	24.0
Gold NPs sonication	111	38.18	2.35	100
	200	44.41	2.03	24.42
	220	64.67	1.44	1.14
	311	77.8	1.22	2.29
Gold NPs conventional	111	38.30	2.34	100
	200	44.53	2.03	29.32
	220	64.87	1.43	2.32
	311	77.67	1.22	2.32

## Data Availability

Not applicable.
